# Acceleration of wound healing using adipose mesenchymal stem cell secretome hydrogel on partial-thickness cutaneous thermal burn wounds: An *in vivo* study in rats

**DOI:** 10.14202/vetworld.2024.1545-1554

**Published:** 2024-07-21

**Authors:** Suryo Kuncorojakti, Awidhan Zainal Adi Pratama, Cahya Asri Antujala, Clarence Theodosius Bernard Harijanto, Rozak Kurnia Arsy, Putut Andika Kurniawan, Yudy Tjahjono, Lucia Hendriati, Teguh Widodo, Ahmad Aswin, Diyantoro Diyantoro, Andi Yasmin Wijaya, Watchareewan Rodprasert, Helen Susilowati

**Affiliations:** 1 Division of Veterinary Anatomy, Department of Veterinary Science, Faculty of Veterinary Medicine, Universitas Airlangga, Surabaya, Indonesia; 2 Research Centre for Vaccine Technology and Development, Institute of Tropical Disease, Universitas Airlangga, Surabaya, Indonesia; 3 Department of Pharmaceutics Faculty of Pharmacy, Widya Mandala Catholic University, Surabaya, Indonesia; 4 Department of Health, Faculty of Vocational Studies, Universitas Airlangga, Surabaya, Indonesia; 5 Veterinary Stem Cell and Bioengineering Innovation Center, Faculty of Veterinary Science, Chulalongkorn University, Bangkok, Thailand

**Keywords:** drug safety, mesenchymal stem cell-conditioned media, stem cell-free-therapy, thermal injury, wound healing

## Abstract

**Background and Aim:**

The intricate healing process involves distinct sequential and overlapping phases in thermal injury. To maintain the zone of stasis in Jackson’s burn wound model, proper wound intervention is essential. The extent of research on the histoarchitecture of thermal wound healing and the application of mesenchymal stem cell (MSC)-free-based therapy is limited. This study aimed to assess the efficacy of MSC-secretome-based hydrogel for treating partial-thickness cutaneous thermal burn wounds.

**Materials and Methods:**

Eighteen male Wistar rats were divided into three groups, namely the hydrogel base (10 mg), hydrogel secretome (10 mg) and Bioplacenton™ (10 mg) treatment groups. All groups were treated twice a day (morning and evening) for 7 days. Skin tissue samples from the animals were processed for histological evaluation using the formalin-fixed paraffin-embedded method on days 3 and 7.

**Results:**

This study’s findings showed that secretome hydrogel expedited thermal burn wound healing, decreasing residual burn area, boosting collagen deposition and angiogenesis, guiding scar formation, and influencing the inflammation response facilitated by polymorphonuclear leukocytes and macrophages.

**Conclusion:**

The secretome hydrogel significantly improves healing outcomes in partial-thickness cutaneous thermal burn wounds. The administration of secretome hydrogel accelerates the reduction of the residual burn area and promotes fibroblast proliferation and collagen density. The repairment of histo-architecture of the damaged tissue was also observed such as the reduction of burn depth, increased angiogenesis and epidermal scar index while the decreased dermal scar index. Furthermore, the secretome hydrogel can modulate the immunocompetent cells by decreasing the polymorphonuclear and increasing the mononuclear cells. Thus, it effectively and safely substitutes for thermal injury stem cell-free therapeutic approaches. The study focuses on the microscopical evaluation of secretome hydrogel; further research to investigate at the molecular level may be useful in predicting the beneficial effect of secretome hydrogel in accelerating wound healing.

## Introduction

Among injuries in humans, burn ranks fourth in frequency. The presence of hypovolemic stress, cardiovascular disease, and other physiological disturbances in burned patients significantly worsens their prognosis and increases the risk of mortality [1]. Some instances of animal burns, despite being rare, still have unfavorable outcomes [2, 3]. Early wound care is necessary to prevent morbidity. This intervention ceases the burn process, minimizes scarring, alleviates pain, forestalls infection and complications, and expedites wound healing [1, 4]. Burn wound healing progresses through the inflammatory, proliferative, and remodeling phases in a sequential and overlapping manner. In the inflammatory stage, neutrophils and monocytes move toward the injury through expanded local blood vessels and extravasation. During inflammation, fibroblasts and keratinocytes activate multiple cytokines and growth factors, marking the onset of the subsequent proliferation phase. During remodeling, biological processes include myofibroblast development, collagen and elastin deposition, wound healing, and scar maturation [5, 6]. As per Jackson’s classification, burn wounds are categorized into three zones: Hyperemia, stasis, and coagulation. The coagulation zone, with complete coagulative necrosis and irreversible tissue loss, lies at the burn’s core, while the periphery hosts the stasis zone, marked by impaired blood flow and potential effectiveness for wound care. Failure to provide adequate early medical intervention could cause irreversible tissue damage, complicate the condition, and prolong the healing process. The hyperemic zone, located at the outermost layer, is characterized by the presence of live cells and vasodilation caused by inflammatory mediators. The treatment method for burns depends on factors such as the degree of burn, size of the affected area, and cause. Burn injuries are treated with topical therapy. Topical therapy is crucial in the early stages of burn injury to prevent contamination or infection [7]. Silver sulfadiazine is the universally accepted standard for burn wound treatment [8]. The solution comprises silver nitrate and sodium sulfadiazine. Sulfadiazine gets released upon silver ion’s attachment to bacterial nucleic acid. Sulfadiazine product reactions hinder microbial metabolism [9]. Silver sulfadiazine’s silver components specifically bind to DNA nucleic acids, inhibiting skin cell multiplication by interacting with structural proteins. Due to the toxic effects of silver on keratinocytes and fibroblasts, its use in treating burn wounds may slow down the healing process [10], necessitating the search for alternative, less harmful methods to expedite burn wound healing [5]. Innovations have been introduced, including stem cells, natural extracts, antibiotics, exosomes, and secretomes [5, 11–16].

Preserving the stasis zone in wound healing depends on the secretome’s ability to generate solubilizing factors essential for the paracrine impact of stem cells. Maintaining intact and salvageable areas during therapy is crucial [17]. The secretomes contain various bioactive molecules such as angiopoietin-1 (ANG-1), platelet-derived growth factor (PDGF), vascular endothelial growth factor (VEGF), hepatocyte growth factor (HGF), fibroblast growth factor (FGF), epidermal growth factor (EGF), interleukins (IL-1, IL-2, IL-4, IL-10, interferon [IFN]-γ, and tumor necrosis factor [TNF]-α), and vesicle-based factors such as exosomes and microvesicles. These molecules have been shown to promote angiogenesis, reduce inflammation, promote re-epithelialization, increase the production of extracellular matrix (ECM), and remodel tissue [15, 18]. Challenges to wound healing through secretome administration persist, specifically concerning stability, delivery, and sustained release. Preserving bioactive molecules within secretomes for controlled release at the wound site is essential. Researchers work on advanced delivery systems, such as hydrogels and nanoparticles, to enhance the effectiveness of topical secretome applications. Hydrogels, being water and hydrophilic polymers, are optimal for topical applications of secretomes in burn wounds. Hydrogels’ three-dimensional structures facilitate gas and ion exchange, provide a cooling sensation, extend bioactive secretome retention, hinder deeper wound penetration, and promote wound healing. Hydrogels facilitate proliferation and angiogenesis in therapeutic applications due to their ability to maintain a moist healing environment. They are less painful, more practical, and simpler to apply and remove [17, 19–21].

The study intends to assess the utility of secretome hydrogel in shaping the histoarchitecture of partial-thickness cutaneous burn wounds. Thus, it can be used as an alternative of an effective treatment for partial-thickness cutaneous burn wound.

## Materials and Methods

### Ethical approval

This study received ethical approval from the Institutional Animal Care and Use Committee of the Faculty of Veterinary Medicine, Universitas Airlangga with certificate number 1.KEH.147.09.2023.

### Study period and location

This study was conducted from June to October 2023 at the Research Center for Vaccine Technology, Institute of Tropical Disease, Universitas Airlangga, Surabaya, Indonesia.

### Mesenchymal stem cell (MSC) secretome preparation

Well-characterized rabbit adipose MSCs (rADMSCs) (positive for CD44, CD90, and CD105, negative for CD45, and with a good capacity in three lineage differentiations, namely osteogenic, chondrogenic, and adipogenic) were obtained from the Research Center for Vaccine Development and Technology, Institute of Tropical Disease, Universitas Airlangga. Rabbit ADMSCs were maintained in α-MEM (α-minimal essential medium) culture media (Gibco, Thermo Fisher Scientific, Grand Island, NY, USA) supplemented with 1% penicillin-streptomycin (Gibco, Thermo Fisher Scientific), 1% Amphotericin B (Gibco, Thermo Fisher Scientific), and 10% fetal bovine serum (Gibco, Thermo Fisher Scientific) at 37°C in a humidified 5% CO_2_ environment. The media were changed every 48 h. For the next 24 h, cells were incubated in serum-free media (starvation method) after achieving 80% confluency. MSC-conditioned media were collected and stored at –80°C for subsequent experiments. This study utilized cells from passages 3 to 6.

The pooled MSC-conditioned media was centrifuged at 4173× *g* for 15 min, then dialyzed against 0.5M phosphate buffer saline (PBS; pH 7.2–7.4) (Gibco, Thermo Fisher Scientific) at 4°C using a 3500 DaMWCO (molecular weight cut off) dialysis tube (Spectra/Por® Dialysis Membrane Tubing, UK) to acquire rADMSC secretomes. PBS was replaced every 24 h with a new one with 4 times. 1M PBS (Gibco, Thermo Fisher Scientific) was used for the fifth replacement. A dialyzed rADMSC secretome was collected, and several growth factors, such as basic FGF (bFGF) (Bioassay Technology Laboratory, Shanghai, China), transforming growth factor beta (TGF-β) (Bioassay Technology Laboratory), VEGF (Bioassay Technology Laboratory), PDGF (Bioassay Technology Laboratory), EGF (Bioassay Technology Laboratory) and insulin-like growth factor (IGF) (Bioassay Technology Laboratory), were quantified by enzyme-linked immunosorbent assay (ELISA). All growth factor quantifications were performed according to the manufacturer’s instructions.

### Hydrogel formulation

Methylparaben (Sigma Aldrich, St. Louis, Missouri, USA) and propylparaben (Sigma Aldrich) were mixed with propylene glycol (Sigma-Aldrich). After a homogenous mixture was obtained, hydroxypropyl methylcellulose (HPMC) (Sigma-Aldrich) as a polymer/gelling agent was added and mixed vigorously. PBS as a control treatment (hydrogel base) was added, while rADMSC secretomes were added to form a secretome hydrogel. For further evaluation, the hydrogels were kept in a refrigerator at 4°C. The hydrogel’s composition is detailed in Table-1.

**Table-1:** Hydrogel formulations.

Ingredients	Concentration (% w/w)	

	Hydrogel base	Secretome hydrogel
Hydroxypropyl methylcellulose	2	2
Propylene glycol	15	15
Methylparaben	0.18	0.18
Propylparaben	0.02	0.02
Phosphate buffer saline	Add 100	-
rADMSC Secretome	-	Add 100

rADMSC=Rabbit adipose mesenchymal stem cell

### Evaluation of MSC secretome hydrogel

This study assessed the hydrogel formulation’s organoleptic qualities, homogeneity, pH level, viscosity, spreading coefficient, and adhesion coefficient.

#### Organoleptic assessment

The hydrogels’ color, consistency, and odor were evaluated during a physical assessment.

#### Homogeneity assessment

To assess the homogeneity of the hydrogel, a pair of transparent glasses was employed. 10 g of hydrogel was placed on the first glass and covered by the second glass. The presence of particles in the hydrogel was observed through visual evaluation.

#### pH measurement

The pH of the hydrogel was measured using a calibrated bench pH meter (OHAUS ST3100-B, Parsippany, New Jersey, USA).

#### Viscosity measurement

A DV1 Viscometer (Brookfield type LV, Middleboro, MA, USA) with spindle number four was employed to assess hydrogel viscosity. 100 mg of hydrogel was placed in a beaker glass, and viscosity was assessed for 10 min at 6 rpm.

#### Spreading coefficient

The spreading coefficient was measured between two transparent glasses. 0.5 g hydrogel was sandwiched between the first and second glasses. 150 g was loaded on the upper part of the second glass and left for 1 min. The calibrated caliper was used to measure the diameter.

#### Adhesion coefficient

The adhesion coefficient was determined using a pair of glasses. 1 g of hydrogel was placed in the first glass and then covered with the second glass. The glasses were pressed with a 1 kg weight for 5 min. An 8 g weight was set on the lower glass’s lower plate. The measurement of the adhesive coefficient was based on the retention time when a pair of glasses was kept intact.

### Experimental animals

Eighteen healthy Wistar male rats, with an average weight of 200 ± 1.87 g, were utilized for this study. At Catholic University Widya Mandala, Surabaya, the animals were kept in the laboratory under a 12-h light-dark cycle after being obtained from PT Biomedical Technology Indonesia in Bogor, Indonesia. In this study, animals were fed a normal chow diet with unlimited access to food and water.

### Partial thickness thermal burn wound model

Animals were acclimatized for 7 days and then divided into three groups (6 animals in each group)before thermal burn wound induction. The animals were anesthetized using 87 mg/kg body weight (BW) of ketamine (Ketamil-Ilium, Troy Laboratories, Glendening, NSW, Australia) and 13 mg/kg BW of xylazine (Interchemie, Venray, Limburg, The Netherlands) before induction. A metal plate with a 20 mm diameter, heated in boiling water for 3 min, was applied to the rat’s back for 10 s to inflict thermal injury. The animals received hydrogel base as a negative control, secretome hydrogel as treatment, and Bioplacenton™ (KalbeMed, Indonesia) for thermal burn wound healing. The daily measurement of burn wounds’ diameters using a caliper determined the residual burn area. The animals were euthanized on days 3 and 7; their skin tissue was collected and then fixed in 10% formalin for histological examination.

### Histological examination

The histological examination was conducted using the formalin-fixed paraffin-embedded tissue protocol. A thin section (4 μm) of skin tissue was stained by hematoxylin and eosin routine staining. The microscopic slides were documented using a Nikon upright microscope Eclipse Ci series with an HD-2 digital camera and NIS Element BR software version 4.30 (Nikon, Tokyo, Japan). The number of fibroblasts, polymorphonuclear leukocytes, tissue macrophages, and new blood vessels (capillaries) were counted under a microscope at a 400-time magnification, whereas histoarchitectural assessments such as collagen density, burn depth, granulation tissue area, dermal thickness, and wounded and normal epithelial thickness were assessed using ImageJ software (https://imagej.net/ij/ij/). The epidermal scar index was calculated by dividing wounded epithelial thickness by normal epithelial thickness, while the dermal scar index was calculated by dividing granulation tissue area by dermal thickness.

### Statistical analysis

The results of this study are presented as bar charts using GraphPad Prism version 9.0 for MacOS (GraphPad Software Inc., California, USA). Parametric statistics were used if the data met the Kolmogorov–Smirnov normality test. Analysis of variance was used in this study to determine the differences between three or more treatment groups, followed by Duncan’s multiple range test. Significance was considered if the p < 0.05. All data analyses were performed using the Statistical Package for the Social Sciences Statistics version 26 (IBM Corp., NY, USA).

## Results

In this study, the secretomes were derived from rADMSCs, and the concentration of growth factors evaluated using ELISA is presented in Table-2. The rADMSC-derived secretomes used in this study contain several growth factors, including bFGF (110.96 ± 17.98 ng/mL), TGF-β (188.15 ± 9.23 μg/L), VEGF (221.56 ± 9.20 μg/L), PDGF (593.57 ± 39.02 ng/mL), EGF (1.12 ± 0.08 μg/L), and IGF (1092.53 ± 189.22 ng/mL).

**Table-2:** Growth factor concentration of rADMSCs secretome.

Growth factor	Concentration	Unit
Basic fibroblast growth factor	110.96 ± 17.98	ng/mL
Transforming growth factor-beta	188.15 ± 9.23	μg/L
Vascular endothelial growth factor	221.56 ± 9.20	μg/L
Platelet-derived growth factor	593.57 ± 39.02	ng/mL
Epidermal growth factor	1.12 ± 0.08	μg/L
Insulin-like growth factor	1092.53 ± 189.22	ng/mL

rADMSC=Rabbit adipose mesenchymal stem cell

According to the evaluation of the hydrogels in this study, both the hydrogel base and secretome hydrogel showed similar characteristics such as clear, semi-solid consistency, odorless, and homogeneous. The pH measurement indicates that both formulations tend to produce a neutral pH ranging from 7.43 ± 0.04 to 7.61 ± 0.02. Similar values were also reported in the viscosity measurement and in the spreading and adhesion coefficient measurements. The details of the hydrogel formulation in both the hydrogel base and secretome hydrogel are presented in Table-3.

**Table-3:** Hydrogel evaluation.

Parameter	Hydrogel base	Secretome hydrogel
Color	Clear	Clear
Consistency	Semi-solid	Semi-solid
Odor	Odorless	Odorless
Homogeneity	Homogenous	Homogenous
pH	7.43 ± 0.04	7.61 ± 0,02
Viscosity	82415.67 ± 801 cps	82515.67 ± 1049 cps
Spreading coefficient	5.3 ± 0.1 cm	5.6 ± 0.1 cm
Adhesion coefficient	2.21 ± 0.12 s	2.39 ± 0.18 s

The residual burn area was determined by measuring the wound diameter through visual observation. From day 1 to day 7, there was a statistically significant difference (p < 0.05). Animals treated with secretome hydrogel and Bioplacenton™ showed improved clinical outcomes than those treated with hydrogel base (Table-4). By Day 7, erythema in the peripheral necrotic crust was still present in the hydrogel base and control groups, while it was absent in both the secretome hydrogel and Bioplacenton™ groups.

**Table-4:** Residual burn area measurement.

Treatment	Residual burn area (cm)							

	Day 0	Day 1	Day 2	Day 3	Day 4	Day 5	Day 6	Day 7
Hydrogel base	2.00 ± 0.00^a^	1.99 ± 0.00^a^	1.98 ± 0.01^a^	1.96 ± 0.01^a^	1.91 ± 0.01^a^	1.86 ± 0.01^a^	1.79 ± 0.01^a^	1.72 ± 0.01^a^
Secretome hydrogel	2.00 ± 0.00^a^	1.97 ± 0.01^b^	1.91 ± 0.01^b^	1.81 ± 0.01^b^	1.71 ± 0.01^b^	1.64 ± 0.01^b^	1.56 ± 0.01^b^	1.53 ± 0.01^b^
Bioplacenton™	2.00 ± 0.00^a^	1.97 ± 0.01^b^	1.91 ± 0.01^b^	1.81 ± 0.01^b^	1.71 ± 0.01^b^	1.64 ± 0.01^b^	1.56 ± 0.01^b^	1.53 ± 0.01^b^

*Different superscripts showed significantly different

**Figure-1:**
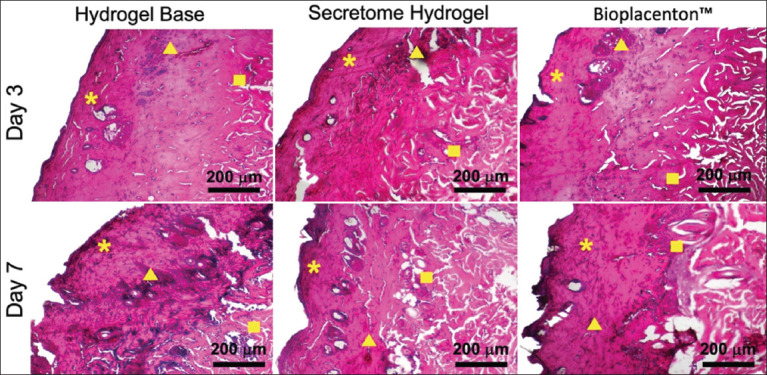
Histological figures of partial thickness cutaneous thermal burn wound. Three different zones, namely zone of coagulation (yellow star), zone of stasis (yellow triangle), and zone of hyperemia (yellow box), were observed in thermal burn wound-induced skin in all conditions on day 3 and day 7.

By day 3, the rat skin’s epidermis was extremely thin under all conditions following a thermal burn wound, while the re-epithelization rate rose significantly by day 7. Three distinct forms of the dermis were observed with unclear demarcation zones (Figure-1).

**Figure-2:**
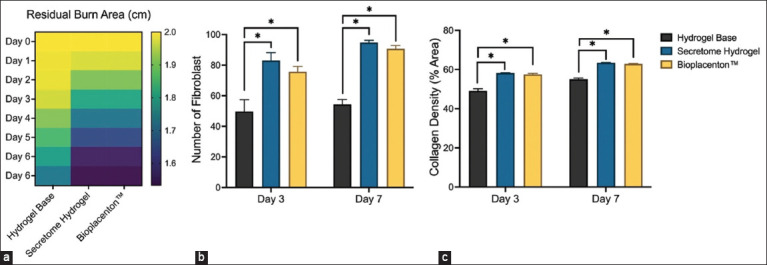
Macroscopic and microscopic evaluation of thermal burn wound. The macroscopic evaluation was based on the residual burn area diameter (a), while the microscopic measurements were based on the number of fibroblasts (b) and collagen density (c) in zone of stasis of the damaged tissue.

A heat map diagram illustrates the macroscopic evaluation of burned skin from day 0 to day 7 (Figure-2a). The group treated with the hydrogel base exhibited slower wound healing. The secretome hydrogel and Bioplacenton™ treatment groups exhibited more advanced wound healing, as indicated by the darker color. On days 3 and 7, the number of fibroblasts was significantly greater (p < 0.05) in both the secretome hydrogel and Bioplacenton™ treatment groups as compared to the hydrogel base-treated animals. On day 7, the fibroblast count was greater than on day 3 (Figure-2b). Figure-2c shows consistent results for collagen density measurement. Animals treated with secretome hydrogel and Bioplacenton™ had denser collagen on days 3 and 7 than those not receiving the treatments.

Histological analysis showed a significant reduction (p < 0.05) in burn tissue depth after treatment with secretome hydrogel. By day 7 post-wounding, animals treated with secretome hydrogel exhibited a significant uptick in new blood vessel formation. The epidermal and dermal scar indices were used to evaluate the extent of thermal wound injury scarring. The scar indices on the epidermis and dermis showed that the secretome hydrogel treatment group had better results than other treatment groups. The epidermis scar index was significantly increased (p < 0.05) in the secretome hydrogel treatment group, while the dermal scar index was decreased.

**Figure-3:**
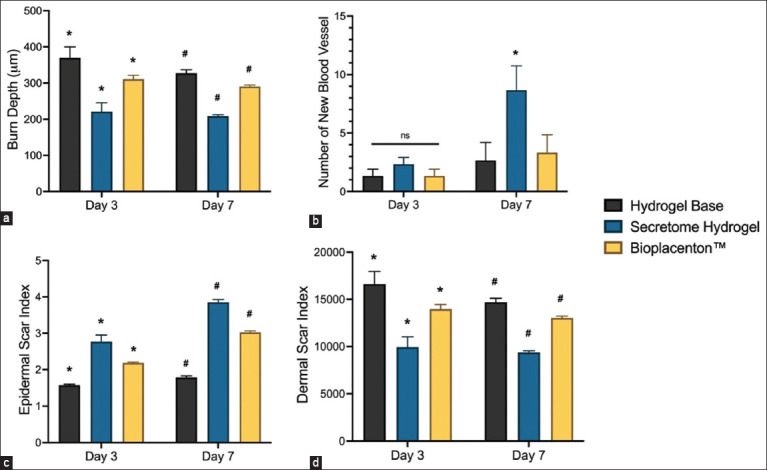
Histoarchitecture evaluation of damaged tissue due to thermal injury. A microscopic-based comprehensive assessment to assess the progression of wound healing including the burn width (a), angiogenesis (b), epidermal (c), and dermal (d) scar indices.

The application of secretome hydrogel led to improved burn recovery and the growth of new blood vessels, as shown in Figures-3a and b. On day 7, an association exists between a decrease in burn thickness and the emergence of new blood vessels in the secretome hydrogel treatment. In the stasis zone of burned skin (Figures-3c and d), the microscopic assessment was conducted to ascertain the count of blood vessels. In rats, the administration of hydrogel results in effective resuscitation and preservation of the status zone.

**Figure-4:**
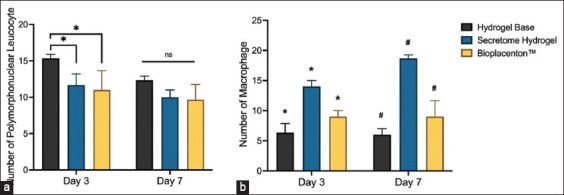
Immunocompetent cell measurement on damaged tissue. The acute and chronic inflammation responses were assessed by calculating the number of polymorphonuclear leukocytes (a) and macrophages (b).

Microscopic assessments were used to evaluate inflammation’s impact on thermal burn wound healing. 3-day post-treatment, polymorphonuclear leukocytes were significantly highest in the untreated group (p < 0.05), while no significant difference was observed among the groups on day 7 (Figure-4a). In the secretome hydrogel group, macrophages significantly increased in number compared to other groups (p < 0.05). The number of macrophages gradually rose from day 3 to day 7 (Figure-4b).

## Discussion

The growth factor profile in this study is similar to the growth factors obtained from placenta-derived MSCs, which can produce bFGF, VEGF, MCP-1, IL-6, and IL-8 [22]. Proteins such as fibronectin, collagen families, and vimentin were identified through a comprehensive study employing a proteomic approach to have significant roles in matrix formation. Important growth factors such as the VEGF families, TGF families, and connective tissue growth factor were also identified [23]. A global standardization of the manufacturing of secretome-derived MSC products is not yet well established. The preparation of the MSC secretomes is primarily performed by centrifuging the expanded medium of MSCs to remove cell debris, waste tissue, and apoptotic bodies [24]. The use of ultrafiltration or dialysis protocol using an ultrafilter unit with MWCO <3500 Dalton to retain the whole conditioned media has also been performed [25]. The filter unit used in this study yielded consistent results. The standardization of secretome-based products is hampered by numerous challenges. From adipose- and amniotic fluid-derived MSCs, there was a difference in molecular factors obtained when investigating solubilized protein yield [26].

A hydrogel formulation based on HPMC was chosen due to its physicochemical properties. Moreover, HPMC is widely used as an excipient in many topical and oral formulations, producing a clear gel that is water soluble, neutral, and stable within the pH range of 3 and 11 [27]. All of the evaluation parameters of both the hydrogel base and secretome hydrogel fulfilled the requirements for a good dermatological preparation [28, 29]. A similar study reporting the evaluation of hydrogel formulation mentioned that a good topical preparation should fulfill several requirements, such as no granules/particles were detected in the gel preparation, homogeneous, pH ranging from 6.5 to 7.6, which did not interfere with the physiological pH of the skin. All of the parameters reported in a previous study by Rahman *et al*. [30] are in agreement with those in this study.

The wound evaluation in this study is consistent with Jackson’s approach to the classification of damaged tissue. In thermal injury, the damaged tissue is characterized by the presence of three different zones: An irreversible zone of coagulation, which is located at the center of the burn with a complete coagulative necrotic mass; a zone of stasis at the periphery of the zone of coagulation with vasoconstricted blood vessels; and a zone of hyperemia with the enlargement of blood vessels due to the local inflammatory response [31]. Thermal injury is classified according to the depth of damage, namely superficial, partial, or full thickness [32]. Burn wound management aims to control the inflammatory response and limit hypertrophic scars [33]. The healing of wounds caused by thermal injury involves a progression of interrelated and overlapping stages: Hemostasis, inflammation, proliferation, and remodeling. Each phase is distinguished by predominant cells capable of initiating multiple biophysical functions [34, 35]. Based on Jackson’s burn wound model, wound management aims to ensure proper resuscitation and safeguard the stasis zone [31].

In tissue damage repair, a highly dynamic cell that plays an important role is called fibroblasts. Fibroblasts are derived from mesenchymal stem and progenitor cells. The migration of progenitor cells to the injury site is controlled by IL-4, IL-13, and IFN-γ [36]. A study in mice showed that the activation of fibroblasts recruits several key signaling pathways, such as PDGF, TGF-β, and Wnt [37]. The Wnt signaling pathway plays an important role in many biological activities of the cell, including proliferation, differentiation, and migration. The cutaneous thermal injury promotes and upregulates Wnt signaling in all stages of the wound healing phase [38]. The number of fibroblasts in the secrotome hydrogel-treated animals increased due to several growth factors, namely bFGF and TGF-β. A similar study in murine models revealed that fibroblast migration was modulated by IL-1β, TGF-β, and bFGF [39]. Furthermore, the use of rat ADMSCs in burn wounds could also promote the secretion of bFGF, VEGF, HGF, and IL-10 [40, 41]. The high number of fibroblasts in the secretome hydrogel treatment in this study had a positive outcome on collagen deposition in the damaged tissue. FGF, VEGF, and TGF-β in the secretome might play an important role by promoting several proteins and glycosaminoglycans involved in ECM formation, such as collagen Type I, II, II, IV, elastin, and metalloproteinases [42]. Induction of fibroblasts by MSCs in matrix production was reported to occur in the proliferation phase [43]. Therefore, both bFGF and collagen matrix could reduce the inflammatory response through the ERK (Extracellular signal-regulated kinases) and TRK (Tropomyosin receptor kinase) pathways [44].

The positive clinical outcome in this study is consistent with a study reporting that adequate wound intervention preserves the zone of stasis [31]. The proangiogenic growth factors, VEGF, TGF-β, PDGF, and bFGF, provided by the MSC secretomes could increase the number of newly formed blood vessels in secretome hydrogel-treated animals. A similar study mentioned that the soluble factors from MSCs such as bFGF, VEGF, placental growth factor, TGF-β, PDGF, ANG-1, IL-6, and monocyte chemotactic protein-1 can promote angiogenesis through a paracrine effect [45]. Other studies also showed that IGF can promote endothelial progenitor cell proliferation, which is also important for angiogenesis [46, 47].

The healing process was assessed by examining scar formation on the epidermis and dermis. On days 3 and 7, the epidermal scar index in the secretome hydrogel group was highest, while the dermal scar index in secretome hydrogel-treated animals was the lowest. During the initial phase of recovery, re-epithelization significantly contributes to the healing process. Several growth factors (HGF, FGF-1, granulocyte colony-stimulating factor [G-CSF], granulocyte-macrophage colony-stimulating factor [GM-CSF], IL-6, VEGF, and TGF-β3) can encourage keratinocytes to function as epithelial precursors [48, 49]. In this study, the presence of VEGF, FGF, and TGF-β in the secretome hydrogel likely contributed to a lower epidermal scar index. This study’s thermal burn induction led to dermal scars, while the application of secretome hydrogel lessened the scar index. MSC soluble factors can attenuate scar formation [46] by inhibiting protein expression of heat shock factor (HSF)-derived nuclear factor kappa B (NF-κB), alpha-smooth muscle actin through the delivery of miR-138-5p to target Sirtuin-1 (SIRT1) and promoting apoptosis of keloid fibroblasts [50].

Inflammation-related cells significantly modulate key healing-signaling pathways like Wnt and TGF-β. The intricate skin microenvironment is regulated significantly by resident immune cells, rendering it a potent first-line defense organ [51]. The animals treated with hydrogel and Biplacenton™ were capable of managing the inflammatory response with fewer polymorphonuclear leukocytes. In a previous study, neutrophil autophagy led to a decrease in polymorphonuclear leukocyte count. Failure to inhibit neutrophil autophagy leads to excessive production of nicotinamide adenine dinucleotide phosphate (NADPH)-mediated reactive oxygen species, resulting in uncontrolled inflammation [52, 53]. The direct influence of MSC secretomes on decreasing polymorphonuclear leukocytes in thermal injury remains elusive. In an ischemic mouse model, protection from brain inflammatory response was achieved by small extracellular vesicles derived from MSC, which decreased the presence of brain leukocytes, specifically polymorphonuclear cells, monocytes, and macrophages [54, 55].

In contrast to polymorphonuclear leukocytes, numerous studies have reported the role of macrophages in wound healing. Thermal injury treated with secretome hydrogel is capable of increasing the number of macrophages. The macrophages in damaged tissue are activated by a set of cytokines (TNF, IL-1, IL-6, IL-8, and IL-12) produced by fibroblasts, lymphocytes, and endothelial cells [56]. This study reveals that profibroblast growth factors in the MSC secretome indirectly activate macrophages. In tissue regeneration, macrophages adopt either pro-inflammatory (M1) or anti-inflammatory (M2) roles [57]. The activation pathway is critical in determining the fate of macrophages. The ratio of M1 and M2 macrophages depends on wound maturation. In the early stage, 85% of macrophages have proinflammatory phenotypes (M1), while during the proliferation phase, the phenotypes change into reparative or anti-inflammatory macrophages (M2). The MSC secretome promoted a macrophage increase from days 3 to 7, correlating with prior research on rat thermal injury inflammation. Macrophage count peaked between days 7 and 14 [33]. The possible mechanism of the positive clinical outcome of the secretome hydrogel-treated animals in thermal injury healing in this study is caused by the polarization of the phenotype into anti-inflammatory (M2) macrophages through signaling pathways such as phosphoinositide 3-kinase-AKT-mammalian target of rapamycin (PI3K/AKT/mTOR), janus kinase-signal transducer and activator of transcription (JAK/STAT), NF-κB, Wnt/β-catenin, and Notch [58].

## Conclusion

A rat model with a partial cutaneous thermal burn wound showed improvement following treatment with secretomes hydrogel. In this study, the MSC secretome was well characterized and produced several growth factors, such as bFGF, TGF-β, VEGF, PDGF, EGF, and IGF, which can accelerate thermal burn wound healing by reducing the residual burn area, promoting collagen deposition and angiogenesis, regulating scar formation, and modulating the inflammation response facilitated by polymorphonuclear leukocytes and macrophages. The hydrogel derived from secretomes could be an efficient and safe alternative for stem cell-free therapy in thermal burn injuries. The single approach of microscopic evaluation to assess the outcome of the utilization of secretome hydrogel might become the limitation of this study. Thus, further research to investigate at molecular level may be useful to predict the beneficial effect of secretome hydrogel in accelerating wound healing.

## Authors’ Contributions

SK and WR: Designed and supervised the study and drafted and revised the manuscript. YT, LH, and TW: Designed and supervised the study. AZAP, CAA, CTBH, RKA, and PAK: Performed the hydrogel formulation, evaluation, and data analysis. AA and HS: Performed ADMSC isolation as well as secretomes production and characterization. DD and AYW: Performed experimental animal and histological assessment. All authors have read, reviewed, and approved the final manuscript.
